# Antagonizing CCR2 With Propagermanium Leads to Altered Distribution of Macrophage Subsets and Favorable Tissue Remodeling After Myocardial Infarction in Mice

**DOI:** 10.1155/cdr/8856808

**Published:** 2025-06-25

**Authors:** Kay Weipert, Holger Nef, Sandra Voss, Jedrzej Hoffmann, Sven Reischauer, Andreas Rolf, Kerstin Troidl, Astrid Wietelmann, Christian W. Hamm, Samuel T. Sossalla, Christian Troidl

**Affiliations:** ^1^Department of Internal Medicine I, Division of Cardiology, University Clinic Giessen, Giessen, Germany; ^2^Department of Experimental Cardiology, Kerckhoff Heart and Thorax Center, Bad Nauheim, Germany; ^3^Max-Planck Institute for Heart and Lung Research, Bad Nauheim, Germany

**Keywords:** CCR2, macrophages, monocytes, myocardial infarction, propagermanium

## Abstract

**Aims:** The aim of the present study was to investigate the inhibition of classically activated macrophages in myocardial infarction (MI) under the influence of the chemokine (C-C motif) receptor 2 (CCR2) antagonist propagermanium (PPG).

**Methods and Results:** Mice (C57BL/6; *n* = 121) were subjected to occlusion of the left anterior descending artery and were randomized to the following groups: (a) MI with daily oral administration of 0.9% sodium chloride (“MI”), (b) MI with oral administration of 8 mg/kg PPG (“MI + PPG”), and (c) sham-operated mice served as control. Mice were euthanized 2, 5, 10, or 21 days after MI for isolation of total RNA, protein, and immunofluorescence measurements. Flow cytometry was performed to investigate peripheral blood leucocytes. Scar size and cardiac function were determined by MRI on Day 7 after surgery and by trichrome staining on Day 21. PPG administration led to a significantly improved ejection fraction (MI + PPG: 38.5% ± 3.4% vs. MI: 23.8% ± 3.0%; *p* < 0.05) after MI. MRI also revealed improved wall thickness (34.7% ± 3.2% vs. 21.8% ± 2.9%; *p* < 0.05) associated with a diminished akinetic area (13.8% ± 4.0% vs. 37.3% ± 5.6%; *p* < 0.01). Trichrome staining confirmed less collagen scar formation in the PPG-treated group (12.7% ± 1.4% vs. 21.9% ± 3.9%; *p* < 0.05). Flow cytometry showed fewer peripheral blood monocytes in MI + PPG than in MI 2 days after treatment (4.0% ± 0.7% vs. 12.7% ± 1.2% of total leucocytes; *p* < 0.05). Immunostaining and western blotting using activation type-specific markers CCR2 and MRC1 demonstrated that the number of alternatively activated macrophages within the infarct zone increased, whereas the overall number was reduced after PPG treatment. PPG led to increased expression of VEGF-*α* and VEGF-*β* in THP-1 cells in vitro and increased capillary density in vivo 2 days after MI (MI-PPG: 1071 ± 81/mm^2^ vs. MI: 648 ± 79/mm^2^ (*p* < 0.05)).

**Conclusion:** Our results suggest that altering the activation type and distribution of invading macrophages in favor of alternative activation improves cardiac remodeling and function following MI.

**Summary**

• First time application of propagermanium in the context of myocardial infarction (MI)
• Time-dependent use of CCR2 antagonism 24 h after MI, allowing initial inflammation
• Evaluation of cardiac function by MRI with extraordinarily large group size
• Correlation of monocytes in circulation and different subsets in the infarct zone
• First time discovery of relation between propagermanium and neoangiogenesis

## 1. Introduction

Myocardial infarction (MI) causes cell death and tissue necrosis and is associated with infiltration of inflammatory cells, primarily neutrophils and monocytes/macrophages, into the infarct area. This so-called inflammatory phase lasts for 1–3 days post MI in mice and represents a crucial process during wound healing as it initiates a complex cascade of molecular, cellular, and physiological responses [1–4]. In this phase, the diminished wall thickness of the infarct area predisposes to rupture of the interventricular septum or left ventricular free wall [5]. In the proliferative phase (3–7 days post MI), granulation tissue is formed and organized by macrophages and activated myofibroblasts, which produce extracellular matrix proteins. This subsequently leads to the maturation phase with the replacement of damaged myocardial tissue by a resilient scar, which is primarily composed of collagen. However, inflammatory processes are not only limited to the infarct area but can also spread to the adjacent border zone of the infarct area, thus affecting healthy myocardial tissue and leading to a deficit in cardiac function; this ultimately constitutes a negative prognostic factor [6–8].

We and others have shown that in a mouse model of MI, different subsets of macrophages appear sequentially in the infarct zone [2, 9, 10]. Early after the initial occlusion, monocytes infiltrate the infarct area and differentiate into macrophages displaying the classical activation pattern (M1). M1 macrophages release cytokines such as interleukin 6 (IL6), interleukin 1-*β* (IL1-*β*), or tumor necrosis factor *α* (TNF-*α*), which account for an inflammatory burst. Furthermore, the release of chemotactic agents by M1 macrophages, including monocyte-chemoattractant protein 1 (MCP-1, also known as CCL2), leads to enhanced recruitment of further circulating leucocytes from the circulation [11]. MCP-1 and its receptor, chemokine (C-C motif) receptor 2 (CCR2), are crucially involved in the recruitment and migration of M1 macrophages into the infarct zone. Furthermore, it has been shown that CCR2 is a specific marker for classical monocytes/macrophages [12]. Also, there is evidence that patients with an increased number of classically activated macrophages in the peripheral blood have a poorer outcome after ST-elevation MI [13].

Given the important role of CCR2 during inflammatory processes after MI, the aim of the present study was to investigate whether blocking CCR2 by the specific antagonist propagermanium (PPG) [14] prevents a long-lasting and overwhelming inflammatory reaction, thereby leading to reduced scar formation and improved myocardial function in a mouse model of MI. PPG, an organogermanium compound, has been shown to effectively interfere with the MCP-1/CCR2 signaling axis, thereby reducing the recruitment of proinflammatory monocytes that contribute to tissue injury and fibrosis. Preclinical studies have demonstrated its efficacy in attenuating atherosclerotic lesion formation [15], reducing renal inflammation and fibrosis [16], and mitigating liver injury in experimental models [17]. Collectively, these findings highlight the therapeutic potential of PPG as a modulator of immune cell recruitment and inflammatory responses across diverse organ systems where MCP-1-driven inflammation is a key pathological feature, warranting further investigation into its clinical applications.

To our knowledge, this is the first time that PPG is investigated in the context of MI.

## 2. Methods

### 2.1. Animal Model

Female mice aged 8 ± 0.9 weeks (mean body weight 25.7 ± 3.5 g; C57BL/6, Elevage Janvier, Le Genest St. Isle, France) were subjected to coronary artery occlusion and randomized to the following groups: (a) coronary artery occlusion combined with daily oral administration of 0.9% sodium chloride (“MI”), (b) coronary artery occlusion combined with daily oral administration of 8 mg/kg body weight PPG (3-[(2-carboxyethyl-oxogermyl)oxy-oxogermyl]propanoic acid) (“MI + PPG”), or (c) sham-operated mice as control (“sham”). Female mice are generally less aggressive than males. This can lead to lower levels of stress-induced variability in cardiovascular measurements, which is particularly important in studies of heart attacks where stress can influence outcomes. Moreover, contrary to the common belief that hormonal fluctuations (such as the estrous cycle) introduce excessive variability, research has shown that the variability in female mice is not significantly greater than in male mice. This means that using female mice can yield reliable and consistent data without the need to control for additional factors related to sex differences. Our intention was to investigate a potential positive clinical effect of CCR2 inhibition in the “most difficult” setting with regard to possible cardioprotective effects of estrogen. A mix of the sexes was no option due to the manageably sized groups to exclude a sex bias and to reduce the number of animals for scientifically statistical results. Since most research in this field is undertaken with male mice, our findings offer additive value.

PPG or sodium chloride was administered once daily by oral gavage starting 24 h after surgery. Mice were euthanized under anesthesia with ketamine (100 mg/kg body weight) and xylazine (12.5 mg/kg body weight) by cervical dislocation and exsanguination 2, 5, 10, or 21 days after MI for isolation of cardiac total RNA or protein and for immunohistochemistry. Mice were subjected to MRI investigations before and 7 days after coronary artery occlusion to assess cardiac functional parameters. Scar formation was investigated by trichrome staining 21 days after surgery.

Mice were sedated by intraperitoneal injection of ketamine (100 mg/kg body weight) and xylazine 2% (12.5 mg/kg body weight). After endotracheal intubation, mice were anesthetized by isoflurane inhalation. Ventilation was accomplished with a rodent ventilator (Hugo Sachs Electronics, Mach, Germany). A thoracotomy was performed at the fourth intercostal space. All muscles overlying the intercostal space were laid open and retracted with 5-0 silk threads; the intercostal muscles were transected. A permanent ligature with a 7-0 Prolene thread (Ethicon, Norderstedt, Germany) was placed around the left anterior descending artery just below the atrioventricular border. Discoloration of the ventricle and typical ECG changes [18] were considered to be evidence of ischemia. Upon separate closure of muscle and skin layers, the lungs unfolded to their original extent. The animals were weaned from the respirator and then extubated. Sham-operated animals were subjected to similar surgery including thoracotomy without the ligature of the coronary artery.

All investigations conform to the Guide for the Care and Use of Laboratory Animals published by the US National Institute of Health [19] and were approved by the appropriate authorities (approval number B2/232 RP Darmstadt, Hessen, Germany).

### 2.2. Magnetic Resonance Imaging

Cardiac MRI measurements were performed on a 7.05 T Bruker PharmaScan equipped with a 300 mT/m gradient system using a custom-built circularly polarized birdcage resonator with a 25-mm inner diameter and the IntraGate self-gating tool (Bruker, Ettlingen, Germany). The 8-week-old mice were subjected to MRI 7 days after MI under anesthesia with 2.0% isoflurane. The measurement is based on the gradient echo method (repetition time 6.2 ms, echo time 2.6 ms, field of view 2.20 × 2.20 cm, slice thickness 1.0 mm, 128 × 128 matrix). The imaging plane was localized using scout images showing the two- and four-chamber view of the heart, and this was followed by acquisition in the short-axis view, orthogonal to the septum in both scouts. Multiple contiguous short-axis slices consisting of seven slices were acquired for complete coverage of the left and right ventricles. MRI data were analyzed using the software Mass4Mice (Medis Medical Imaging Systems BV, Leiden, Netherlands). Scar size, measured by MRI, was defined as akinetic area, as previously described [20].

### 2.3. Histological Analysis

Frozen sections (6 *μ*m) were prepared using a cryostat (Leica CM 1950, Leica Microsystems, Bensheim, Germany), transferred to silane-coated slides, and fixed in 4% paraformaldehyde for 5 min. After rinsing in phosphate-buffered saline, they were incubated with the primary antibody against *α*-SMA-FITC (Sigma-Aldrich Chemie GmbH, Munich, Germany), CD68 (AbD Serotec, Kidlington, United Kingdom), MRC1 (CD206) Alexa Fluor 488 (BioLegend, San Diego, California, United States), CCR2 (Abbiotec, San Diego, California, United States), and CD31-PE (BD Pharmingen, Heidelberg, Germany). Incubation with secondary antibodies (anti-mouse-Cy3 (Chemicon, Hofheim, Germany), anti-rabbit-cy3 (Chemicon), or biotinylated anti-rabbit IgG (Dianova, Hamburg, Germany)) was followed by incubation with streptavidin-Cy3 or streptavidin-Cy2 (Rockland Immunochemicals, Philadelphia, United States). Nuclei were stained by DAPI (Invitrogen, Groningen, Netherlands) or Draq5 (Axxora, Lörrach, Germany). The sections were visualized using a Leica fluorescence microscope (DM4000B) or confocal laser scanning microscope (SP5, both from Leica, Wetzlar, Germany). Incubation with phosphate-buffered saline instead of the first antibody was used to visualize nonspecific staining. The perivascular immunopositive cells within the infarct zone were counted in all sections. CD68^+^/CD206^+^ cells were classified as M2 activated macrophages and CD68^+^/CD206^−^ cells as M1 macrophages.

Masson trichrome staining (Sigma-Aldrich) was carried out according to the manufacturer's instructions and was used for histological quantification of scar formation using ImageJ 1.46 software (NIH Image, Bethesda, Maryland, United States) as described previously [21]. Infarct scar area is expressed as a proportion of the total area of the left ventricle.

### 2.4. FACS Analysis

The distribution of peripheral blood leucocytes was assessed by flow cytometry (FACS) using an antibody against CD11b. Briefly, peripheral blood samples (50 *μ*L) were obtained by retro-orbital puncture, immediately transferred into FACS tubes, and stained for 30 min at 4°C with fluoroisothiocyanate (FITC)-linked anti-CD11b antibody (ImmunoTools, Friesoythe, Germany). Prior to FACS, red blood cells were lysed in the tubes by adding 1 mL ammonium chloride–based lysing buffer (BD Biosciences GmbH, Heidelberg, Germany) and washed twice with phosphate-buffered saline. Data acquisition was performed with a flow cytometer (FACScan BD Biosciences) using CellQuest Pro Software (BD Biosciences). At least 30,000 leucocyte events were acquired in a single measurement. Data was analyzed using FlowJo software (V 9.1 for Macintosh, Tree Star Inc., Ashland, Oregon, United States). The following leucocyte subpopulations were distinguished based on immunophenotype and light-scattering properties: granulocytes (SSC^high^CD11b^+^), monocytes (SSC^low^CD11b^+^), and lymphocytes (SSC^low^CD11b^−^).

### 2.5. Western Blotting

Isolation of total protein and subsequent western blotting were performed as previously described [22]. Protein concentrations were measured by the method of Lowry et al. [23]. Proteins (20 *μ*g) were separated by electrophoresis on 4%–12% Bis-Tris polyacrylamide gels (Invitrogen, Groningen, Netherlands) and subsequently transferred to nitrocellulose membranes (Invitrogen). For primary immunodetection, specific antibodies against CCR2 (Abbiotec) and MRC1 (BioLegend) were used. After incubation with the secondary peroxidase-labeled anti-rabbit (Abcam, Cambridge, United Kingdom) or anti-donkey (GE Healthcare, Buckinghamshire, United Kingdom) antibody, the membrane was photographed by a CCD imager (ChemiDoc XRS, Bio-Rad, Munich, Germany), and the relative amounts of proteins were quantified using QuantityOne software (Bio-Rad).

### 2.6. Cell Culture

THP-1 monocytes were purchased from the German collection of microorganisms and cell cultures (DSMZ). Cell culture was carried out at 37°C and 5% CO_2_ in a 95% humidified atmosphere (Heraeus Cytoperm, Heraeus Holding GmbH, Hanau, Germany). Cells were cultured in RPMI 1640, 20% fetal calf serum, 1% glutamine, and 10 mM penicillin/streptomycin (c.c. pro GmbH, Neustadt, Germany). Cells were seeded into six-well plates (Greiner bio-one GmbH, Frickenhausen, Germany) at a density of 5 × 10^5^ cells per milliliter to obtain optimal exponential growth curves. Doubling times were 35–50 h.

Cells were incubated for 3 h with 100 ng/mL MCP-1 (R&D Systems, Minneapolis, Minnesota, United States) or 1000 ng/mL PPG (Alfa Aesar GmbH, Karlsruhe, Germany) or the combination of PPG and MCP-1. Following incubations, cells were harvested and stored at −80°C in freezing medium (70% RPMI 1640, 20% FCS, 10% DMSO; c.c. pro GmbH, Neustadt Germany) at 4 × 10^6^ cells/mL.

### 2.7. Quantitative Real-Time PCR

After thawing, 4 × 10^6^ THP-1 cells were suspended in 350 *μ*L RLT buffer (Qiagen GmbH, Hilden, Germany) and 10 *μ*L *β*-mercaptoethanol (Sigma-Aldrich). Total RNA isolation was carried out using the RNeasy Micro Kit (Qiagen), and this was followed by DNase treatment (Turbo DNA-free, Ambion Inc., Austin, Texas, United States). The concentration and quality of isolated RNA were evaluated by a spectrophotometer (Nanodrop ND-1000, PEQLAB Biotechnologie GmbH, Erlangen, Germany). Gene-specific RT-PCR primers were selected using the sequences from the PrimerBank database [4] or designed using the Primer3 online tool (http://biotools.umassmed.edu/bioapps/primer3_www.cgi). qRT-PCR was conducted as previously described [9]. In brief, qRT-PCR was performed using the 7500 real-time PCR system by Applied Biosystems (Foster City, California, United States). In a 25-*μ*L reaction, 100 nM primer, 1× IQ SYBR Green Supermix, and 1 *μ*L cDNA (1:10) were mixed. Assays were performed in triplicate. The relative amount of target vascular endothelial growth factor (VEGF) mRNA (VEGF-*α* sense: 5⁣′-TCTACCTCCACCATGCCAAGT, antisense: 5⁣′-GCTGCGCTGATAGACATCCA-3⁣′; VEGF-*β* sense: 5⁣′-CAGAGGAAAGTGGTGTCATGGA-3⁣′, antisense: 5⁣′-ACCGGATCATGAGGATCTGCA-3⁣′) was normalized to GAPDH (sense: 5⁣′-TGGGCTACACTGAGCACCAG-3⁣′, antisense: 5⁣′-CAGCGTCAAAGGTGGAGGAG-3⁣′). The delta-Ct (threshold cycle) was used to calculate the fold change in mRNA abundance versus a baseline control, which was set to 1.

### 2.8. Data Analysis

Data are reported as mean ± standard error of the mean (SEM). All statistical analyses were performed using GraphPad Prism Version 10.1.1 for Mac (GraphPad Software, La Jolla, California, United States). D'Agostino and Pearson omnibus normality tests were used to test for normal distribution. Statistical analyses were performed with Student's *t*-test for unpaired samples and normally distributed datasets. Statistical comparisons of more than two experimental groups were performed with one-way ANOVA followed by the Bonferroni–Holmes test. The Kruskal–Wallis one-way ANOVA was used for datasets that did not pass the normality test. Survival curves were constructed by using the Kaplan–Meier method. The log-rank test (Mantel–Cox) was used to compare the survival curves. Values of *p* < 0.05 were considered to be statistically significant.

## 3. Results

### 3.1. Survival Rate and Infarct Size

**Figure 1:**
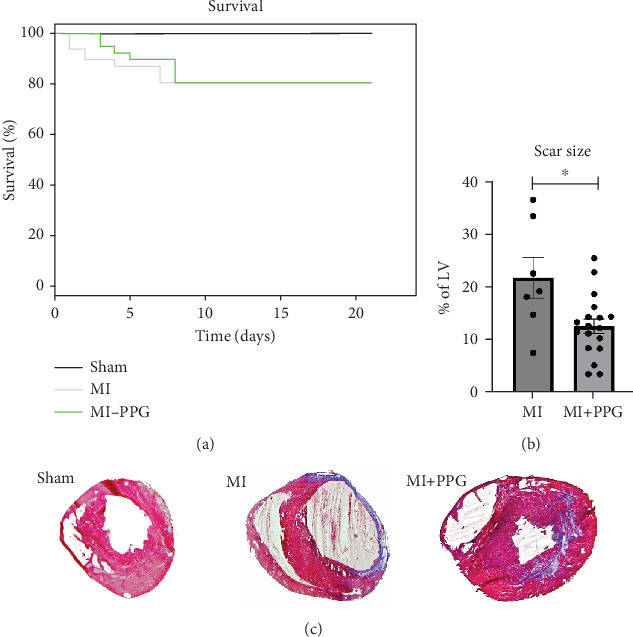
Survival rate and histological evaluation of scar size by trichrome staining 21 days after myocardial infarction. (a) Kaplan–Meier 21-day survival rates in mice after myocardial infarction (*n* = 68). (b) Portion of scar in the total left ventricular area (percentage) is less in PPG-treated mice versus MI; ⁣^∗^*p* < 0.05. (c) Example of a trichrome-stained infarct area. Images are taken at a twofold magnification. Blue represents collagen, pink represents viable myocardium.

During a maximum follow-up period of 21 days, there were no significant differences in survival rates between untreated and treated animals: 7 of 49 untreated mice died and 7 of 48 PPG-treated mice died. All deaths occurred within the first 8 days after coronary artery occlusion. None of the sham-treated mice died during the experimental period (Figure 1a).

Trichrome staining of explanted hearts showed a smaller scar size after occlusion in PPG-treated mice than in those that underwent occlusion without treatment (MI + PPG 12.7% ± 1.4% area fraction vs. MI 21.9% ± 3.9% area fraction; *p* < 0.05; *n* = 25 total) (Figure 1).

### 3.2. Functional Myocardial Parameters

**Figure 2:**
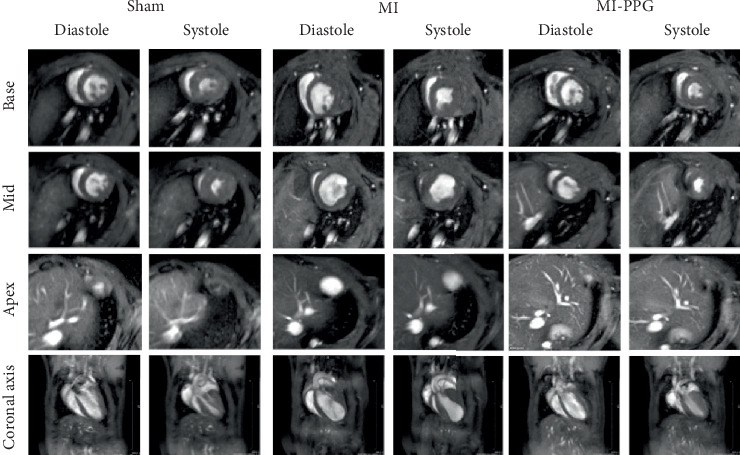
Cardiac MR imaging. Exemplary cardiac MR images 7 days after MI. Images show diastole and systole in axial planes from base to apex and coronal axis.

**Figure 3:**
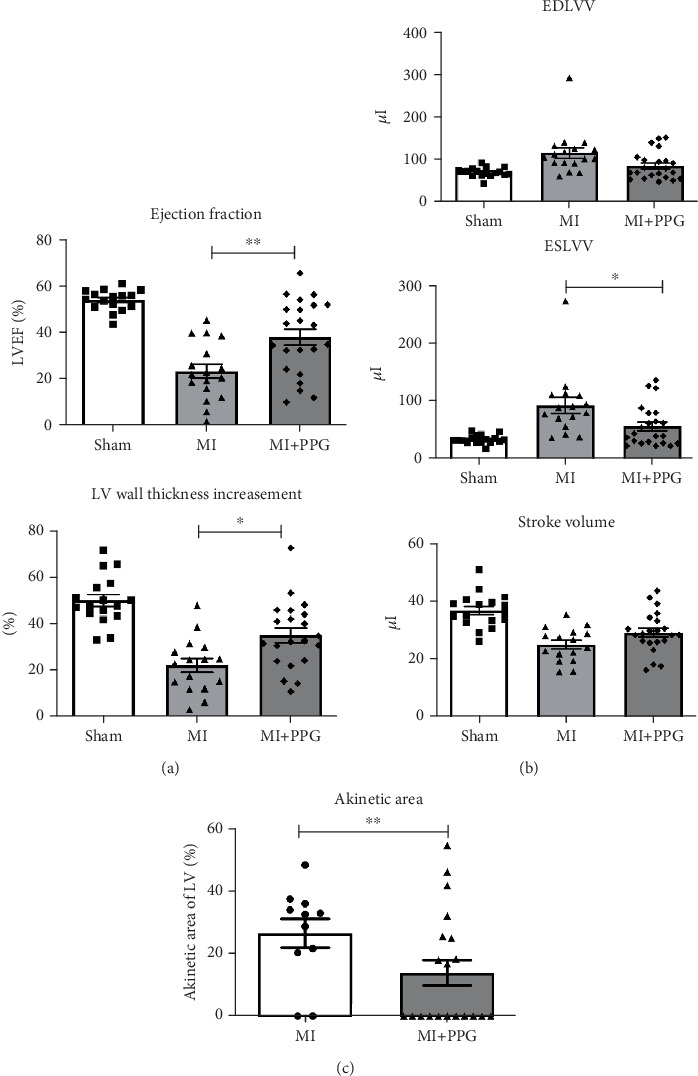
Functional cardiac parameters assessed by MRI 7 days after myocardial infarction. (a) Application of PPG led to a higher left ventricular ejection fraction (EF) and enhanced wall thickness increasement; ⁣^∗∗^*p* < 0.01, ⁣^∗^*p* < 0.05. (b) End-diastolic left ventricular volume (EDLVV) and stroke volume did not differ significantly between groups, but the end-systolic volume was significantly lower in the MI + PPG group; ⁣^∗^*p* < 0.05. (c) The akinetic area of the total left ventricle was reduced after PPG treatment; ⁣^∗^*p* < 0.05.

MRI (Figure 2) demonstrated a mean ejection fraction (EF) of 54.3% ± 1.1% in all mice prior to MI. The EF after MI was markedly higher in MI + PPG mice (38.5% ± 3.4%; *n* = 22) than in MI mice (23.8 ± 3.0; *n* = 17) after 7 days (*p* < 0.05). Accordingly, left ventricular wall thickening during contraction was enhanced in PPG-treated mice (MI-PPG 34.68% ± 3.16% vs. MI 21.82% ± 2.93%; *p* < 0.05) (Figure 3). Left ventricular volumes also differed: the end-diastolic left ventricular volume (EDLVV) was significantly higher (*p* < 0.01) in the MI group (115.1 ± 12.5* μ*L) than in the sham group (69.4 ± 2.6* μ*L), whereas there was no significant difference in this parameter between PPG-treated mice (84.6 ± 7.0* μ*L) and sham-treated mice. Enlarged ventricular size consistently led to augmented end-systolic left ventricular volume (ESLVV). The mice in the MI group showed a significantly higher ESLVV than those in the sham or PPG-MI groups (MI 91.6 ± 13.9* μ*L vs. sham 32.7 ± 1.8* μ*L; *p* < 0.001; vs. PPG-MI 55.6 ± 7.6* μ*L; *p* < 0.05) (Figure 3). The analysis of regional akinetic wall motion, which is determined in order to assess the scar size [20], revealed a smaller akinetic area in the MI-PPG group than in the MI group (MI-PPG: 13.8% ± 4.0% vs. MI: 37.3% ± 5.6%; *p* < 0.01) (Figure 3). These findings correlate with the histological results (Figure 1(b)).

### 3.3. Leucocyte Distribution in the Peripheral Blood

**Figure 4:**
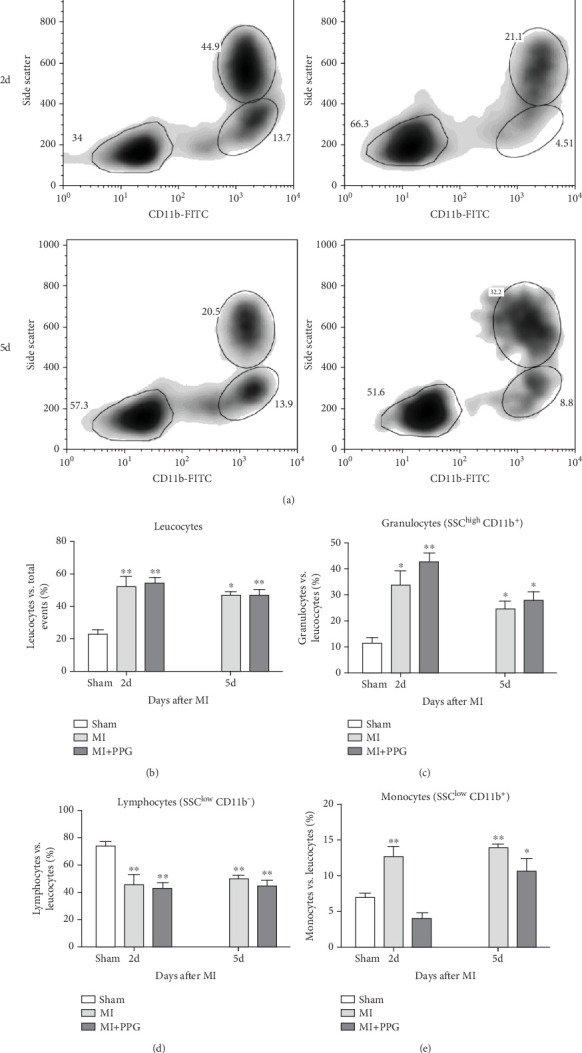
FACS analysis. (a) Flow cytometric assessment of circulating peripheral leucocytes in mice 2 (2d) and 5 (5d) days after MI compared with sham-operated animals. Leucocyte subpopulations were distinguished based on their immunophenotypical and light-scattering properties: granulocytes (SSC^high^CD11b^+^), monocytes (SSC^low^CD11b^+^), and lymphocytes (SSC^low^CD11b^−^). (b) The relative amount of leucocytes was higher in both MI groups 2 and 5 days after MI versus sham; ⁣^∗∗^*p* < 0.01. (c) Likewise, the proportion of granulocytes was higher 2 and 5 days after MI in both groups versus sham; ⁣^∗^*p* < 0.05, ⁣^∗∗^*p* < 0.01. (d) The proportion of lymphocytes was significantly lower at both time points in both groups versus sham; ⁣^∗∗^*p* < 0.01. (e) Whereas monocytes were significantly higher after 2 days in the MI group (⁣^∗∗^*p* < 0.01), there was no significant difference in the relative number of monocytes in the PPG group versus sham. After 5 days, however, both MI groups had higher monocyte levels versus sham; ⁣^∗^*p* < 0.05, ⁣^∗∗^*p* < 0.01; each group *n* = 5.

In order to investigate the effects of CCR2 antagonism on circulating blood leucocytes, FACS analysis was performed 2 and 5 days after MI (Figure 4a). In both experimental groups, a significantly greater proportion of total leucocytes was observed 2 days after surgery compared with the sham group (sham: 22.5% ± 2.1% vs. MI: 48.9% ± 3.2% vs. MI-PPG: 50.4% ± 3.7%; *p* < 0.01; each group *n* = 5). At 5 days, leucocyte levels were still significantly higher, showing a slight but insignificant regression (MI: 45.8% ± 3.0% [*p* < 0.05] vs. MI-PPG: 46.6% ± 3.7% [*p* < 0.01]) (Figure 4b). In parallel, the proportion of circulating granulocytes was also higher after MI than in the sham group, especially after 2 days (sham: 11.54% ± 2.1%, MI: 29.2% ± 4.5% [*p* < 0.05]; MI-PPG: 35.1% ± 7.4% [*p* < 0.01]) (Figure 4c). Granulocytes dropped considerably on the fifth postsurgical day (MI: 24.7% ± 2.8% vs. MI-PPG: 27.7% ± 3.6%). The higher proportion of granulocytes was associated with a significantly lower (*p* < 0.01) proportion of lymphocytes after MI at both time points investigated, with no significant differences between the two experimental groups (2d: MI: 45.9% ± 6.4% and MI-PPG: 43.5% ± 3.2%; 5d: MI: 49.7% ± 3.8% and MI-PPG: 45.1% ± 4.2% vs. sham: 73.8% ± 2.9%) (Figure 4d). Thus, no significant changes were observed in the total number of leucocytes or the proportion of granulocytes and lymphocytes between MI and MI-PPG groups at either time point investigated.

Findings concerning the proportions of monocytes revealed a different scenario. Two days after MI, the proportion of monocytes in the untreated MI group was significantly higher than that of the sham group. In contrast, the proportion of monocytes in mice with PPG treatment was even slightly lower than that of the sham animals (MI 12.7% ± 1.2% vs. MI-PPG 4.0% ± 0.7% vs. sham group 6.9% ± 0.4%). Thus, the results 2 days after MI displayed a striking difference between PPG-treated mice and the untreated group (*p* < 0.001). After 5 days, there were no significant differences between both experimental groups (MI 13.9% ± 0.5% vs. MI-PPG 10.6% ± 2.0%) (Figure 4e).

### 3.4. Macrophages Within the Infarct Zone

**Figure 5:**
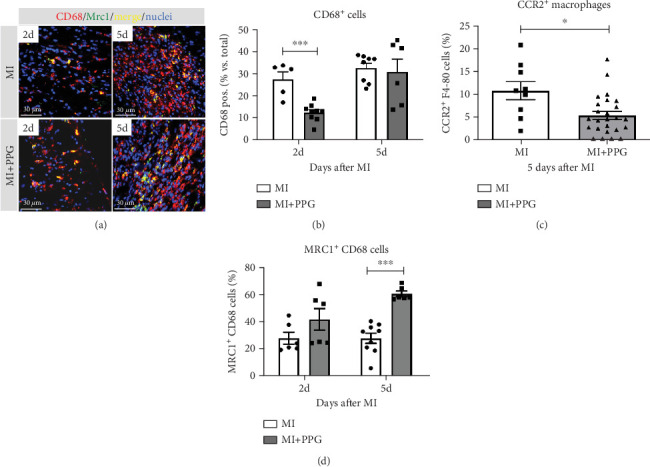
Immunohistological analysis of the infarct zone. (a) Staining within the infarct zone 2 and 5 days after MI with CD68 (red), MRC1 (green), and DAPI (blue). Cardiac tissue from the MI + PPG group had a significantly higher proportion of alternatively activated macrophages (MRC1-pos. MAC) after 5 days; ⁣^∗∗∗^*p* < 0.001, MI + PPG versus MI (d). (b) Two days after MI, the relative number of macrophages (CD68-positive cells) was lower in the MI + PPG group than in the MI group; ⁣^∗∗∗^*p* < 0.001. (c) Five days after MI, the proportion of classically activated macrophages (CCR2-positive cells) was lower in MI + PPG hearts versus MI; ⁣^∗^*p* < 0.05.

**Figure 6:**
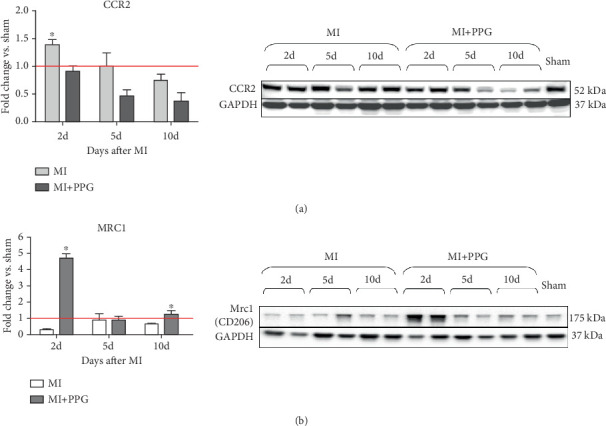
Western blot analysis of macrophage activation profile. (a) The relative amount of CCR2 protein was significantly higher 2 days after MI in the MI group than in the MI + PPG group; ⁣^∗^*p* < 0.05. (b) MRC1 protein levels were significantly higher 2 and 10 days after MI in the MI + PPG group than in the MI group; ⁣^∗^*p* < 0.05 versus MI; each group *n* = 8. The red line depicts the relative level of protein expression in sham animals.

To investigate whether the lower number of circulating monocytes also influenced the number of monocytes/macrophages that migrated into the infarct zone, macrophages were immunohistochemically quantified after staining with the specific pan-macrophage antigen CD68 [24] (Figure 5a). Within the infarct zone, a markedly lower proportion of macrophages (CD68-positive cells) in PPG-treated mice was observed after 2 days compared with the proportion in untreated mice (MI-PPG: 12.2% ± 1.3% vs. MI: 27.3% ± 3.5%; *p* < 0.001; each *n* = 8) (Figure 5b). MI + PPG mice also showed fewer M1 macrophages within the infarct zone after 2 days (MI-PPG 5.4% ± 0.8% vs. MI 10.8% ± 1.9%; *p* < 0.05; each *n* = 8) (Figure 5c). Furthermore, costaining with CD68 and MRC1, a marker for M2 macrophages [25], revealed that antagonism of CCR2 led to a shift of macrophage subpopulations within the infarct zone toward alternative activation. After 2 days, a trend, yet insignificant difference was apparent (MI-PPG: 41.6% ± 7.9% vs. MI: 27.6% ± 4.4%; *p* = 0.06); after 5 days, the difference was highly significant (MI-PPG: 60.4 ± 2.0 vs. MI: 27.6 ± 3.7; *p* < 0.001) (Figure 5d). Both results were confirmed by western blot analysis using CCR2- and MRC1-specific antibodies. The amount of CCR2 protein within the infarct zone in the MI-PPG group was slightly lower than that in the MI group at all time points and reached statistical significance after 2 days (2d: MI-PPG 0.90 ± 0.10 fold change vs. control (FC) vs. MI 1.38 ± 0.10 FC; *p* < 0.05; 5d: MI-PPG 0.46 ± 0.10 FC vs. MI 0.99 ± 0.25 FC; 10d: MI-PPG FC 0.36 ± 0.16 vs. MI FC 0.74 ± 0.10) (Figure 6a). In parallel, the amount of MRC1 protein was significantly higher in PPG-treated mice than in mice of the MI group 2 and 10 days after permanent coronary vessel occlusion (2d: MI-PPG 3.30 ± 1.19 FC vs. MI 0.49 ± 0.14 FC [*p* < 0.05]; 5d: MI-PPG 0.70 ± 0.34 FC vs. MI 1.15 ± 0.30 FC [*p* = 0.15]; 10d: MI-PPG 1.20 ± 0.18 FC vs. MI 0.60 ± 0.13 FC [*p* < 0.05]; each group *n* = 8) (Figure 6b).

### 3.5. Neovascularization

**Figure 7:**
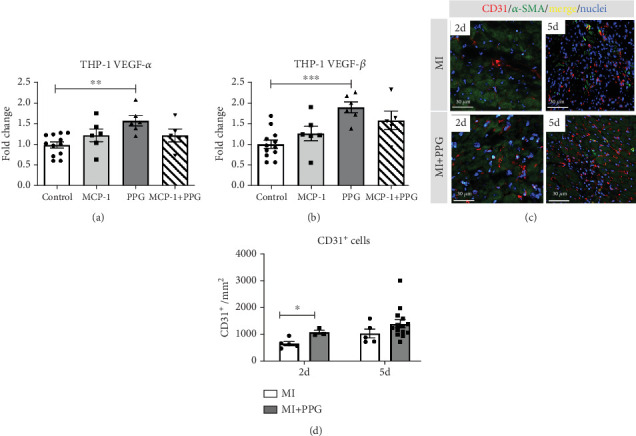
Neovascularization. (a, b) PPG treatment resulted in increased VEGF-*α* and VEGF-*β* expression in THP-1 cells in vitro, ⁣^∗∗^*p* < 0.01 and ⁣^∗∗∗^*p* < 0.001 versus control. (c, d) In vivo staining within the infarct zone 2 and 5 days after MI for CD31 (red), *α*-smooth muscle actin (*α*-SMA, green), and DAPI (blue). MI + PPG hearts had greater capillary density 2 days after MI; ⁣^∗^*p* < 0.05 versus MI.

Aside from simple diffusion, neovascularization is the only known mechanism that can supply the hypoxic infarct zone with essential nutrients; it can also facilitate cell migration into the infarcted tissue (inflammatory cells as well as myofibroblasts and other cells). MCP-1 is a well-known inducer of angiogenesis [26]; therefore, our aim was to examine whether PPG interferes with this signaling. We first studied THP-1 cells in vitro and analyzed the expression pattern of VEGF-*α* and VEGF-*β* after exposure to MCP-1, PPG, or both versus control (sodium chloride administration). PPG had a significant positive amplifying impact on VEGF-*α* expression (1.58 ± 0.12 FC; *p* < 0.01) and VEGF-*β* expression (1.88 ± 0.13 FC; *p* < 0.001), each *n* = 6 (Figure 7a,b). Immunohistological investigations in vivo confirmed these findings and showed a significantly higher capillary density, measured with anti-CD31 staining (Figure 7c,d), in PPG-treated animals after 2 days and still a slight although insignificant difference after 5 days (2d: MI-PPG 1071 ± 81/mm^2^ vs. MI 648 ± 79/mm^2^ [*p* < 0.05]; 5d: MI-PPG 1387 ± 151/mm^2^ vs. MI 1026 ± 168/mm^2^ [*p* = 0.06]; each group *n* = 8).

## 4. Discussion

Antagonism of the chemokine receptor CCR2 has been investigated in various fields of experimental cardiology and vascular medicine, including inhibition of restenosis [27, 28], diet-induced insulin resistance [17, 29], and hypertensive atherosclerosis [30, 31]; here, we examined its possible applicability to post-MI therapy. The aim of this study was to investigate the consequences of CCR2 antagonism 24 h after MI, allowing the organism a full inflammatory response for 1 day. Here, we demonstrate for the first time that interfering with the recruitment of classically activated inflammatory monocytes by inhibition of CCR2 with PPG is associated with increased capillary density, improved cardiac function, and a smaller myocardial scar.

Inflammatory processes following MI play an important role in post-MI remodeling. A certain level of inflammatory activity seems to be crucial for the recruitment of inflammatory cells and fibroblasts as well as phagocytosis of apoptotic and necrotic cells, but excessive inflammation leads to the expansion of the infarct zone to nonischemic neighboring tissue. Anti-inflammatory therapy with corticosteroids after MI has thus far shown promising rather than harmful effects in clinical studies and corresponding meta-analysis [32]. Moreover, glucocorticoids are a well-known trigger that stimulates monocytes to differentiate into alternatively activated macrophages [33]. Nevertheless, interference in macrophage recruitment patterns must be performed in a targeted and careful manner. Preclinical [34–36] and clinical data [37, 38] suggest that insufficient or excessive monocyte recruitment affects the outcome after MI adversely, leading to an increased infarct size, left ventricular dilatation, and ultimately heart failure. It is controversial whether monocytosis is just a common manifestation of large myocardial infarcts or whether it leads to further infarct enlargement due to an excessive proinflammatory reaction; there may be a bidirectional association between large infarcts and monocytosis.

Distinct subsets of monocytes, such as classically and alternatively activated monocytes, occur within the circulation. The role of different subsets of macrophages in wound healing after MI has been recently described [2, 9, 10, 39]. CCR2 is abundantly expressed on circulating inflammatory monocytes, but also tissue resident CCR2− and CCR2+ macrophages differentially orchestrate monocyte recruitment and fate specification following myocardial injury [12, 40]. Early after ischemia, an innate immune response within the affected myocardium leads to recruitment of inflammatory cells, including monocytes, from the circulation. These monocytes differentiate into macrophages in the infarct area [41] and show a classical activation profile. In addition to inflammatory cytokines such as TNF-*α*, IL1-*β*, IL6, and MCP-1 are released, leading to further recruitment of monocytes and other leucocytes via the MCP-1/CCR2 axis [42, 43].

The impact of specific oral antagonism of CCR2, for example, by PPG, and the subsequent effects on classical monocyte activation after MI might be of utmost importance because of its central role in immune response. CCR2-knockout mice after MI have yielded attenuated left ventricular remodeling [44, 45]. Similar effects were observed after monocyte-directed RNA interference targeting CCR2 [46]. On the other hand, overexpression of MCP-1 leads to an excessive inflammatory response and may be related to interstitial myocardial fibrosis and ultimately heart failure [47]. In our mouse model of MI, the administration of PPG led to an enhanced LVEF. Another conclusive diagnostic parameter is the size of the myocardial scar: histological and imaging methods both demonstrated a consistently smaller scar in the PPG-treated group. Differences in absolute infarct size are most likely due to differences in the two methods used, as trichrome staining detects only the actual collagen scar tissue, whereas MRI detects the akinetic area and includes impaired but not necessarily devitalized myocardial tissue, thus leading to higher values of total infarct size [48]. While a smaller scar and better myocardial function are encouraging signs, they do not automatically guarantee improved survival. Several factors that might explain this disconnect in this particular case are among others an arrhythmogenic risk. Even with reduced scar size, the border zone between healthy and injured tissue can remain electrically unstable. This heterogeneity may promote life-threatening arrhythmias that can lead to sudden cardiac death. This argument is supported by the fact that we rarely found cardiac rupture in the dead animals.

By FACS analysis, we show for the first time the influence of PPG on circulating leucocytes following MI. PPG proved to be an effective CCR2 antagonist by significantly reducing the numbers of monocytes within the circulation after 2 days of treatment. Since in PPG-treated mice the number of other leucocytes, including lymphocytes and granulocytes, was unaltered compared with untreated animals after MI, PPG effects can be regarded as specific for monocytes. It has already been shown that CCR2 signaling induces monocyte egress from the bone marrow [49] and that MI activates CCR2-positive hematopoietic stem and progenitor cells [50]. Hence, it is quite likely that the decreased proportion of inflammatory macrophages in the infarct zone reflects on the one hand the reduction of circulating monocytes due to inhibited release from the bone marrow and on the other hand accounts for impaired migration into the infarcted myocardium. From this, one can conclude that reduced proportions of total inflammatory monocytes within the circulation cause a reduction of classically activated macrophages within the infarct zone.

The immunohistological findings are in accordance with the FACS results, which revealed a significantly lower total CD68^+^ cell count 2 days after MI in the PPG-treated group than in the untreated group. In the circulation as well as within the infarct zone, there were no significant differences detected in the total monocyte cell population between the control group and the PPG-treated group at a later time point (5 days). However, the proportion of MRC1-positive macrophages (alternative activation) was still higher 5 days after MI in PPG-treated mice. A higher level of total MRC-1 protein in PPG-treated mice than in untreated mice was overall confirmed by western blotting. Discrepant to our immunohistological findings, in our study, we did not demonstrate a higher protein concentration of MRC-1 after 5 days by western blotting but only after 2 and 10 days. Immunofluorescence is more accurate when it comes to describing specific changes in cellular subsets. Western blotting is prone to capturing Mrc1 expression from a mixed population of cells. It is common knowledge that CD206 is mostly expressed on monocytes/macrophages and dendritic cells. But Cuartero et al. have also shown that 31% of so-called N2 neutrophils can express CD206/MRC1 [51]. Thus, background expression levels in whole tissue assays might dilute the signal detected in a purified macrophage population. Another explanation might be a heterogeneity in the inflammatory response among individual subjects after MI, which could contribute to variable results between whole tissue versus isolated cell analyses due to limited group size.

CCR2 levels were significantly lower in the PPG group. These observations are in line with the concept that a persistent inflammatory response within the infarct zone, induced by classical inflammatory macrophages, leads to an impaired reparative function of M2 macrophages [52]. A targeted experimental depletion of M2 macrophages has shown to be associated with a reduced cardiac function and an increased scar size [53]. On the other hand, small extracellular vesicles derived from M2 macrophages have been associated with improved cardiac repair post MI [54].

Macrophages are a heterogeneous cell population with multiple functions. Alternatively activated macrophages in particular seem to play a substantial role in extracellular matrix remodeling and angiogenesis [55–59]. Our investigation is the first to report the surprising result that PPG appears to promote neoangiogenesis. MCP-1 itself is a well-known promoter of angiogenesis [60–62], and antagonism of CCR2 is regarded to be a new potential therapeutic approach to suppress choroidal neovascularization [63]. Here, however, we demonstrate that PPG is a stronger inducer of VEGF in vitro than MCP-1, an observation that was confirmed immunohistologically by increased capillary density in vivo within the infarct zone 2 days after MI. One could speculate that either PPG is a partial agonist at CCR2 or it acts through a different receptor and pathway. The fact that in vitro VEGF induction by PPG is higher than that caused by MCP-1 alone contradicts the first hypothesis and is supported by the finding that PPG does not act by direct CCR2 inhibition but rather via GPI-anchored proteins in the proximity of the CCR2 receptor [14]. The exact molecular mechanism of the proangiogenic effects of PPG remains unclear and requires further investigation.

Interestingly, it has been shown that PPG is able to form complexes with adrenaline [64]; thus, improved cardiac function with PPG may be due to a reduction in sympathetic signaling, which is an established therapeutic approach in heart failure and MI and may provide evidence of possible pleiotropic mechanisms of action of PPG.

Our study demonstrates that interfering with the molecular profile and distribution of monocytes/macrophages during the remodeling process after MI might be an interesting and promising therapeutic concept. Partial suppression of the MCP-1/CCR2 axis using PPG leads to a milder inflammatory response shortly after coronary occlusion. In the course of ensuing remodeling processes, the mild inflammation leads to reduced scar size, improved cardiac function, and increased neovascularization. As an outlook, it remains imperative to further elucidate the molecular mechanisms underlying these observations. Future investigations should not only address how alterations in macrophage subsets directly contribute to the remodeling process but also incorporate additional mechanistic studies. In this context, employing mobility shift assays could reveal changes in transcription factor binding dynamics that regulate macrophage polarization, while binding assays would be instrumental in characterizing direct interactions between PPG and key signaling molecules involved in macrophage function. Such comprehensive analyses would provide deeper insight into the specific processes driving cardiac remodeling and help establish a more detailed mechanistic framework for PPG's therapeutic effects.

## 5. Limitation

The differentiation of leucocytes from our FACS analysis is mainly based on CD11b presence and difference in side scatter properties. Markers like CD45, Ly6G, or Ly6C might have further helped in accurate discrimination of myeloid cells.

The current strategy of using CCR2 for M1 and MRC1 for M2 macrophages was based on prior studies that linked these markers to proinflammatory and anti-inflammatory states, respectively. However, we acknowledge that macrophage polarization is a complex and dynamic process and that these markers alone may not fully capture the heterogeneity of immune cell populations in myocardial tissue postinfarction. To address this, further flow cytometry experiments in myocardial tissue are needed to distinguish infiltrated leucocytes and to study the effect of PPG on the expression of polarization markers of M1 and M2 macrophages and their corresponding cytokines.

## Data Availability

The data that support the findings of this study are available from the corresponding author upon reasonable request.

## References

[1] Frangogiannis N. G., Smith C. W., Entman M. L. (2002). The Inflammatory Response in Myocardial Infarction. *Cardiovascular Research*.

[2] Nahrendorf M., Swirski F. K., Aikawa E. (2007). The Healing Myocardium Sequentially Mobilizes Two Monocyte Subsets With Divergent and Complementary Functions. *Journal of Experimental Medicine*.

[3] Frangogiannis N. G. (2006). The Mechanistic Basis of Infarct Healing. *Antioxidants & Redox Signaling*.

[4] Wang X., Seed B. (2003). A PCR Primer Bank for Quantitative Gene Expression Analysis. *Nucleic Acids Research*.

[5] Gao X. M., Xu Q., Kiriazis H., Dart A. M., Du X. J. (2005). Mouse Model of Post-Infarct Ventricular Rupture: Time Course, Strain- and Gender-Dependency, Tensile Strength, and Histopathology. *Cardiovascular Research*.

[6] Nathan C. (2002). Points of Control in Inflammation. *Nature*.

[7] Frangogiannis N. G., Ren G., Dewald O. (2005). Critical Role of Endogenous Thrombospondin-1 in Preventing Expansion of Healing Myocardial Infarcts. *Circulation*.

[8] Abbate A., Bonanno E., Mauriello A. (2004). Widespread Myocardial Inflammation and Infarct-Related Artery Patency. *Circulation*.

[9] Troidl C., Mollmann H., Nef H. (2009). Classically and Alternatively Activated Macrophages Contribute to Tissue Remodelling After Myocardial Infarction. *Journal of Cellular and Molecular Medicine*.

[10] Rizzo G., Gropper J., Piollet M. (2023). Dynamics of Monocyte-Derived Macrophage Diversity in Experimental Myocardial Infarction. *Cardiovascular Research*.

[11] Imhof B. A., Aurrand-Lions M. (2004). Adhesion Mechanisms Regulating the Migration of Monocytes. *Nature Reviews. Immunology*.

[12] Geissmann F., Jung S., Littman D. R. (2003). Blood Monocytes Consist of Two Principal Subsets With Distinct Migratory Properties. *Immunity*.

[13] Abo-Aly M., Shokri E., Chelvarajan L., Tarhuni W. M., Tripathi H., Abdel-Latif A. (2023). Prognostic Significance of Activated Monocytes in Patients With ST-Elevation Myocardial Infarction. *International Journal of Molecular Sciences*.

[14] Yokochi S., Hashimoto H., Ishiwata Y. (2001). An Anti-Inflammatory Drug, Propagermanium, May Target GPI-Anchored Proteins Associated With an MCP-1 Receptor, CCR2. *Journal of Interferon & Cytokine Research*.

[15] Yamashita T., Kawashima S., Ozaki M. (2002). Propagermanium Reduces Atherosclerosis in Apolipoprotein E Knockout Mice via Inhibition of Macrophage Infiltration. *Arteriosclerosis, Thrombosis, and Vascular Biology*.

[16] Kitagawa K., Wada T., Furuichi K. (2004). Blockade of CCR2 Ameliorates Progressive Fibrosis in Kidney. *American Journal of Pathology*.

[17] Mulder P., van den Hoek A. M., Kleemann R. (2017). The CCR2 Inhibitor Propagermanium Attenuates Diet-Induced Insulin Resistance, Adipose Tissue Inflammation and Non-Alcoholic Steatohepatitis. *PLoS One*.

[18] Wehrens X. H., Kirchhoff S., Doevendans P. A. (2000). Mouse Electrocardiography: An Interval of Thirty Years. *Cardiovascular Research*.

[19] Institute of Laboratory Animal Resources (US) (1986). *Committee on Care, and Use of Laboratory Animals. Guide for the care and use of laboratory animals. No. 86*.

[20] Schneider J. E., Wiesmann F., Lygate C. A., Neubauer S. (2006). How to Perform an Accurate Assessment of Cardiac Function in Mice Using High-Resolution Magnetic Resonance Imaging. *Journal of Cardiovascular Magnetic Resonance: Official Journal of the Society for Cardiovascular Magnetic Resonance*.

[21] Takagawa J., Zhang Y., Wong M. L. (2007). Myocardial Infarct Size Measurement in the Mouse Chronic Infarction Model: Comparison of Area- and Length-Based Approaches. *Journal of Applied Physiology*.

[22] Troidl C., Nef H., Voss S. (2010). Calcium-Dependent Signalling Is Essential During Collateral Growth in the Pig Hind Limb-Ischemia Model. *Journal of Molecular and Cellular Cardiology*.

[23] Lowry O. H., Rosebrough N. J., Farr A. L., Randall R. J. (1951). Protein Measurement With the Folin Phenol Reagent. *Journal of Biological Chemistry*.

[24] Pulford K. A., Sipos A., Cordell J. L., Stross W. P., Mason D. Y. (1990). Distribution of the CD68 Macrophage/Myeloid Associated Antigen. *International Immunology*.

[25] Porcheray F., Viaud S., Rimaniol A. C. (2005). Macrophage Activation Switching: An Asset for the Resolution of Inflammation. *Clinical and Experimental Immunology*.

[26] Goede V., Brogelli L., Ziche M., Augustin H. G. (1999). Induction of Inflammatory Angiogenesis by Monocyte Chemoattractant Protein-1. *International Journal of Cancer*.

[27] Usui M., Egashira K., Ohtani K. (2002). Anti-Monocyte Chemoattractant Protein-1 Gene Therapy Inhibits Restenotic Changes (Neointimal Hyperplasia) After Balloon Injury in Rats and Monkeys. *FASEB Journal*.

[28] Liehn E. A., Piccinini A. M., Koenen R. R. (2010). A New Monocyte Chemotactic Protein-1/Chemokine CC Motif Ligand-2 Competitor Limiting Neointima Formation and Myocardial Ischemia/Reperfusion Injury in Mice. *Journal of the American College of Cardiology*.

[29] Tamura Y., Sugimoto M., Murayama T. (2010). C-C Chemokine Receptor 2 Inhibitor Improves Diet-Induced Development of Insulin Resistance and Hepatic Steatosis in Mice. *Journal of Atherosclerosis and Thrombosis*.

[30] Ishibashi M., Hiasa K., Zhao Q. (2004). Critical Role of Monocyte Chemoattractant Protein-1 Receptor CCR2 on Monocytes in Hypertension-Induced Vascular Inflammation and Remodeling. *Circulation Research*.

[31] Chan C. T., Moore J. P., Budzyn K. (2012). Reversal of Vascular Macrophage Accumulation and Hypertension by a CCR2 Antagonist in Deoxycorticosterone/Salt-Treated Mice. *Hypertension*.

[32] Giugliano G. R., Giugliano R. P., Gibson C. M., Kuntz R. E. (2003). Meta-Analysis of Corticosteroid Treatment in Acute Myocardial Infarction. *American Journal of Cardiology*.

[33] Vallelian F., Schaer C. A., Kaempfer T. (2010). Glucocorticoid Treatment Skews Human Monocyte Differentiation Into a Hemoglobin-Clearance Phenotype With Enhanced Heme-Iron Recycling and Antioxidant Capacity. *Blood*.

[34] Panizzi P., Swirski F. K., Figueiredo J. L. (2010). Impaired Infarct Healing in Atherosclerotic Mice With Ly-6C (hi) Monocytosis. *Journal of the American College of Cardiology*.

[35] Maekawa Y., Anzai T., Yoshikawa T. (2004). Effect of Granulocyte-Macrophage Colony-Stimulating Factor Inducer on Left Ventricular Remodeling After Acute Myocardial Infarction. *Journal of the American College of Cardiology*.

[36] Leuschner F., Rauch P. J., Ueno T. (2012). Rapid Monocyte Kinetics in Acute Myocardial Infarction Are Sustained by Extramedullary Monocytopoiesis. *Journal of Experimental Medicine*.

[37] Tsujioka H., Imanishi T., Ikejima H. (2009). Impact of Heterogeneity of Human Peripheral Blood Monocyte Subsets on Myocardial Salvage in Patients With Primary Acute Myocardial Infarction. *Journal of the American College of Cardiology*.

[38] Aoki S., Nakagomi A., Asai K. (2011). Elevated Peripheral Blood Mononuclear Cell Count Is an Independent Predictor of Left Ventricular Remodeling in Patients With Acute Myocardial Infarction. *Journal of Cardiology*.

[39] Kadl A., Meher A. K., Sharma P. R. (2010). Identification of a Novel Macrophage Phenotype That Develops in Response to Atherogenic Phospholipids via Nrf2. *Circulation Research*.

[40] Bajpai G., Bredemeyer A., Li W. (2019). Tissue Resident CCR2- and CCR2+ Cardiac Macrophages Differentially Orchestrate Monocyte Recruitment and Fate Specification Following Myocardial Injury. *Circulation Research*.

[41] van Furth R., Cohn Z. A. (1968). The Origin and Kinetics of Mononuclear Phagocytes. *Journal of Experimental Medicine*.

[42] Frangogiannis N. G. (2008). The Immune System and Cardiac Repair. *Pharmacological Research*.

[43] Frangogiannis N. G. (2004). The Role of the Chemokines in Myocardial Ischemia and Reperfusion. *Current Vascular Pharmacology*.

[44] Kaikita K., Hayasaki T., Okuma T., Kuziel W. A., Ogawa H., Takeya M. (2004). Targeted Deletion of CC Chemokine Receptor 2 Attenuates Left Ventricular Remodeling After Experimental Myocardial Infarction. *American Journal of Pathology*.

[45] Hayasaki T., Kaikita K., Okuma T. (2006). CC Chemokine Receptor-2 Deficiency Attenuates Oxidative Stress and Infarct Size Caused by Myocardial Ischemia-Reperfusion in Mice. *Circulation Journal: Official Journal of the Japanese Circulation Society*.

[46] Majmudar M. D., Keliher E. J., Heidt T. (2013). Monocyte-Directed RNAi Targeting CCR2 Improves Infarct Healing in Atherosclerosis-Prone Mice. *Circulation*.

[47] Kolattukudy P. E., Quach T., Bergese S. (1998). Myocarditis Induced by Targeted Expression of the MCP-1 Gene in Murine Cardiac Muscle. *American Journal of Pathology*.

[48] Ojha N., Roy S., Radtke J. (2008). Characterization of the Structural and Functional Changes in the Myocardium Following Focal Ischemia-Reperfusion Injury. *American Journal of Physiology. Heart and Circulatory Physiology*.

[49] Serbina N. V., Pamer E. G. (2006). Monocyte Emigration From Bone Marrow During Bacterial Infection Requires Signals Mediated by Chemokine Receptor CCR2. *Nature Immunology*.

[50] Dutta P., Sager H. B., Stengel K. R. (2015). Myocardial Infarction Activates CCR2(+) Hematopoietic Stem and Progenitor Cells. *Cell Stem Cell*.

[51] Cuartero M. I., Ballesteros I., Moraga A. (2013). N2 Neutrophils, Novel Players in Brain Inflammation After Stroke. *Stroke*.

[52] Nahrendorf M., Pittet M. J., Swirski F. K. (2010). Monocytes: Protagonists of Infarct Inflammation and Repair After Myocardial Infarction. *Circulation*.

[53] Leblond A. L., Klinkert K., Martin K. (2015). Systemic and Cardiac Depletion of M2 Macrophage Through CSF-1R Signaling Inhibition Alters Cardiac Function Post Myocardial Infarction. *PLoS One*.

[54] Li L., Cao J., Li S. (2023). M2 Macrophage-Derived sEV Regulate Pro-Inflammatory CCR2(+) Macrophage Subpopulations to Favor Post-AMI Cardiac Repair. *Advanced Science*.

[55] Schnoor M., Cullen P., Lorkowski J. (2008). Production of Type VI Collagen by Human Macrophages: A New Dimension in Macrophage Functional Heterogeneity. *Journal of Immunology*.

[56] Hunt T. K., Knighton D. R., Thakral K. K., Goodson W. H., Andrews W. S. (1984). Studies on Inflammation and Wound Healing: Angiogenesis and Collagen Synthesis Stimulated In Vivo by Resident and Activated Wound Macrophages. *Surgery*.

[57] Leibovich S. J., Polverini P. J., Shepard H. M., Wiseman D. M., Shively V., Nuseir N. (1987). Macrophage-Induced Angiogenesis Is Mediated by Tumour Necrosis Factor- *α*. *Nature*.

[58] Jetten N., Verbruggen S., Gijbels M. J., Post M. J., De Winther M. P., Donners M. M. (2014). Anti-Inflammatory M2, but Not Pro-Inflammatory M1 Macrophages Promote Angiogenesis In Vivo. *Angiogenesis*.

[59] Barbay V., Houssari M., Mekki M. (2015). Role of M2-Like Macrophage Recruitment During Angiogenic Growth Factor Therapy. *Angiogenesis*.

[60] Niu J., Azfer A., Zhelyabovska O., Fatma S., Kolattukudy P. E. (2008). Monocyte Chemotactic Protein (MCP)-1 Promotes Angiogenesis via a Novel Transcription Factor, MCP-1-Induced Protein (MCPIP). *Journal of Biological Chemistry*.

[61] Hong K. H., Ryu J., Han K. H. (2005). Monocyte Chemoattractant Protein-1-Induced Angiogenesis Is Mediated by Vascular Endothelial Growth Factor-A. *Blood*.

[62] Capoccia B. J., Gregory A. D., Link D. C. (2008). Recruitment of the Inflammatory Subset of Monocytes to Sites of Ischemia Induces Angiogenesis in a Monocyte Chemoattractant Protein-1-Dependent Fashion. *Journal of Leukocyte Biology*.

[63] Xie P., Kamei M., Suzuki M. (2011). Suppression and Regression of Choroidal Neovascularization in Mice by a Novel CCR2 Antagonist, INCB3344. *PLoS One*.

[64] Nakamura T., Shimada Y., Takeda T., Sato K., Akiba M., Fukaya H. (2015). Organogermanium Compound, Ge-132, Forms Complexes With Adrenaline, ATP and Other Physiological cis-Diol Compounds. *Future Medicinal Chemistry*.

